# Caspase-1 inhibition: mechanistic insights and comprehensive review of reported inhibitors to date

**DOI:** 10.1007/s10787-026-02361-9

**Published:** 2026-08-21

**Authors:** Asif Khan, Atta Ullah, Maira Gabriela Paetzold, Flavio Augusto Vicente Seixas

**Affiliations:** 1 Laboratory of Structural Biochemistry, Department of Technology, State University of Maringa, Umuarama, PR 87506-370 Brazil; 2 Department of Zoology, Institute of Biosciences, University of São Paulo, São Paulo, SP Brazil

**Keywords:** Caspase-1, Inflammasome, NLRP3, Cytokines, Inflammation, Inhibitors

## Abstract

Caspase-1 is a key protease that regulates inflammation by mediating the maturation of pro-inflammatory cytokines IL-1β and IL-18 as well as inducing the pyroptotic cell death. Dysregulation in caspase-1 activity is implicated in various inflammatory, autoimmune and metabolic disorders. This review systematically analyzes natural, synthetic and computationally predicted caspase-1 inhibitors, emphasizing their structural, mechanistic and pharmacological characteristics. We provide data on cellular and enzymatic inhibition, including IC₅₀ values when they are available and list the compounds that have moved on to preclinical or clinical trials. Selective inhibition of caspase-1 remains challenging because the catalytic domain and substrate-binding pocket are highly conserved among caspase family, which makes it challenging to design specific inhibitors. Although, numerous candidates reported exhibit promising caspase-1 inhibitory activities, however, there is still a lack of research regarding caspase–1-specific inhibitors, and further studies are required to investigate novel and effective caspase-1 inhibitors, through in vivo, in vitro, and in silico approaches and their cytotoxicity, bioavailability and solubility.

## Introduction

Inflammation is a fundamental biological response that protects the host by eliminating pathogens, clearing damaged or dead cells and cellular debris and promoting tissue repair to restore homeostasis (Rathinam and Chan 2018). However, any dysregulation of the inflammatory response contributes to the development of several diseases, including autoimmune disorders, neurodegeneration complications, metabolic disorders and cancer (Dhani et al. 2021). Inflammation can be broadly classified as acute or chronic. Acute inflammation is a self-limiting, rapid and the body respond very quickly that develops within hours to days following infection/ tissues injury; however, chronic inflammation persists for months and/ or even years, owing to unresolved inflammatory stimuli or impaired resolution mechanisms (Chopra et al. 2024). Persistent chronic inflammation promotes tissue damage and is strongly associated with the clinical pathogenesis of cancer, autoimmune diseases and neurological abnormalities (Nasef et al. 2017). To avoid such disorders, it’s therefore essential to understand the exact mechanism underlying inflammation in order to develop more targeted therapies to mitigate these conditions.

Inflammation is a highly coordinated physiological process regulated by a wide range of chemical mediators and signaling molecules that actively orchestrate innate immune responses through well-organized chemical pathways (Rubin et al. 2018). Central to these cellular pathways is the caspase family, a specialized group of cysteine-dependent aspartate-specific proteases that regulate inflammation and programmed cell (White 1999). Caspases contain a catalytic cysteine residue in their active site that cleaves substrate proteins specifically after aspartic acid residues (Cade and Clark 2015). Among the inflammatory caspases, caspase-1 is the central prototypical enzyme responsible for coordinating innate immune responses. However, upon inflammasome activation, caspase-1 prototypically processes the inactive precursors pro-IL-1β and pro-IL-18 into their mature and biologically active form i.e. IL-1β and IL-18, thereby promoting inflammatory signaling and host defense against infection (Lopategi et al. 2018). Similarly, in addition to the cytokine’s maturation, the activated caspase-1 also activates gasdermin D, leading to membrane pore formation and induction pyroptosis, a highly inflammatory form of programmed cell death (Denes et al. 2012; Molla et al. 2020).

Caspase-1 is activated through multiprotein complexes called inflammasomes, which assemble when in response to cellular stress, damage-associated molecular pattern (DAMPs) and/or pathogen-associated molecular pattern (PAMPs) (Amarante-Mendes et al. 2018). Inflammasome assembly the recruitment and autocatalytic activation of caspase-1, enabling the proteolytic maturation of the pro-inflammatory cytokines (pro-IL-1β and pro-IL-18) and the induction of pyroptosis (Lamkanfi et al. 2007). Consequently, caspase-1 serves is a key and central effector of inflammasome signaling coordinating innate immune responses through cytokines maturation and the initiation of pyroptotic cell death. The biological effects of the caspase-1/inflammasome axis are highly context-dependent, contributing to the host defense, pathogen clearance and tissue repair through controlled inflammatory responses, whereas excessive or sustained activation can drive chronic inflammations like, tissue injuries and the development of different inflammatory complications. Conversely, the overactivation of caspase-1 may impair cytokines production, weaken antimicrobial immunity and delay tissues repair (Amarante-Mendes et al. 2018; Chopra et al. 2024).

Given its central role in regulating inflammation and pyroptosis, aberrant and/or impaired regulation of caspase-1, is associated with several inflammatory and immune-mediated disorders (Sun and Ou 2026). Consequently, caspase-1 has emerged as an attractive and potential therapeutic target for the treatment of diseases driven by dysregulated inflammasome signaling (Garrido et al. 2019; Molla et al. 2020). Accordingly, considerable effort has been devoted to identifying compounds capable of modulating the caspase-1-mediated pathway. These compounds may act either by directly inhibiting caspase-1 enzymatic activity and/or indirectly by targeting the upstream components of inflammasome signaling pathways, thereby reducing caspase-1 activation and subsequent maturation of IL-1β and IL-18, respectively (Garrido et al. 2019; Dhani et al. 2021).

### Inflammatory caspases

In the recent years, several promising plant-derived compounds have been investigated for their potential to modulate caspase-1-medidate inflammatory responses, highlighting natural products as a valuable source of lead molecules for inflammatory drug discovery (Watanabe and Lam 2004). However, these compounds exhibit diverse mechanisms of action. For example, some of them directly inhibit the enzymetic activity of caspase-1, many act indirectly by modulating upstream inflammasome signaling pathways or regulating caspase-1 expression, ultimately reducing the maturation of cytokines (Tőzsér and Benkő 2016). In addition to natural products, synthetic and biologically derived compounds have also demonstrated therapeutic potential as a modulator of caspase-1 signaling, however, these candidates are substantially differing in their mechanism of action, efficacy and pose negative consequences on living systems/human health (Randle et al. 2001; Caruso et al. 2022).

Therefore, this review aims to provide a comprehensive mechanistic perspective on caspase-1 activation and inhibition, compiling evidences from computational, experimental and preclinical studies to guide the development of novel anti-inflammatory therapeutics.

## Methodology

The systematic search of the literature on caspase-1 inhibitors was performed by searching the relevant papers using the major scientific sources, such as PubMed, ScienceDirect and Google Scholar (June, 2025). The keywords used included the caspase-1 inhibitors, inhibition of inflammasome, NLRP3 and caspase-1 and IL-1β or IL-18 inhibition. The criteria for inclusion in this review were based on studies that provided direct evidences regarding inhibition of caspase-1 or modulations of NLRP3 inflammasome pathway as evident from effects on caspase-1 activity or on IL-1β and IL-18 generation *via* experimental and computational methods. Studies that were not mechanistically supportive, non-peer reviewed articles and duplicate references were not included in this review.

Further literature sources were used to validate the data about enzymes such as the BRENDA enzyme database (Hauenstein et al. 2026) and to find the compounds that affect caspase-1. For example, Google and ResearchGate were only used for additional searches and getting full texts. This strategy aimed at providing transparency of the process of literature selection. (Hauenstein et al. 2026)

### Caspases: classifications and biology

**Fig. 1:**
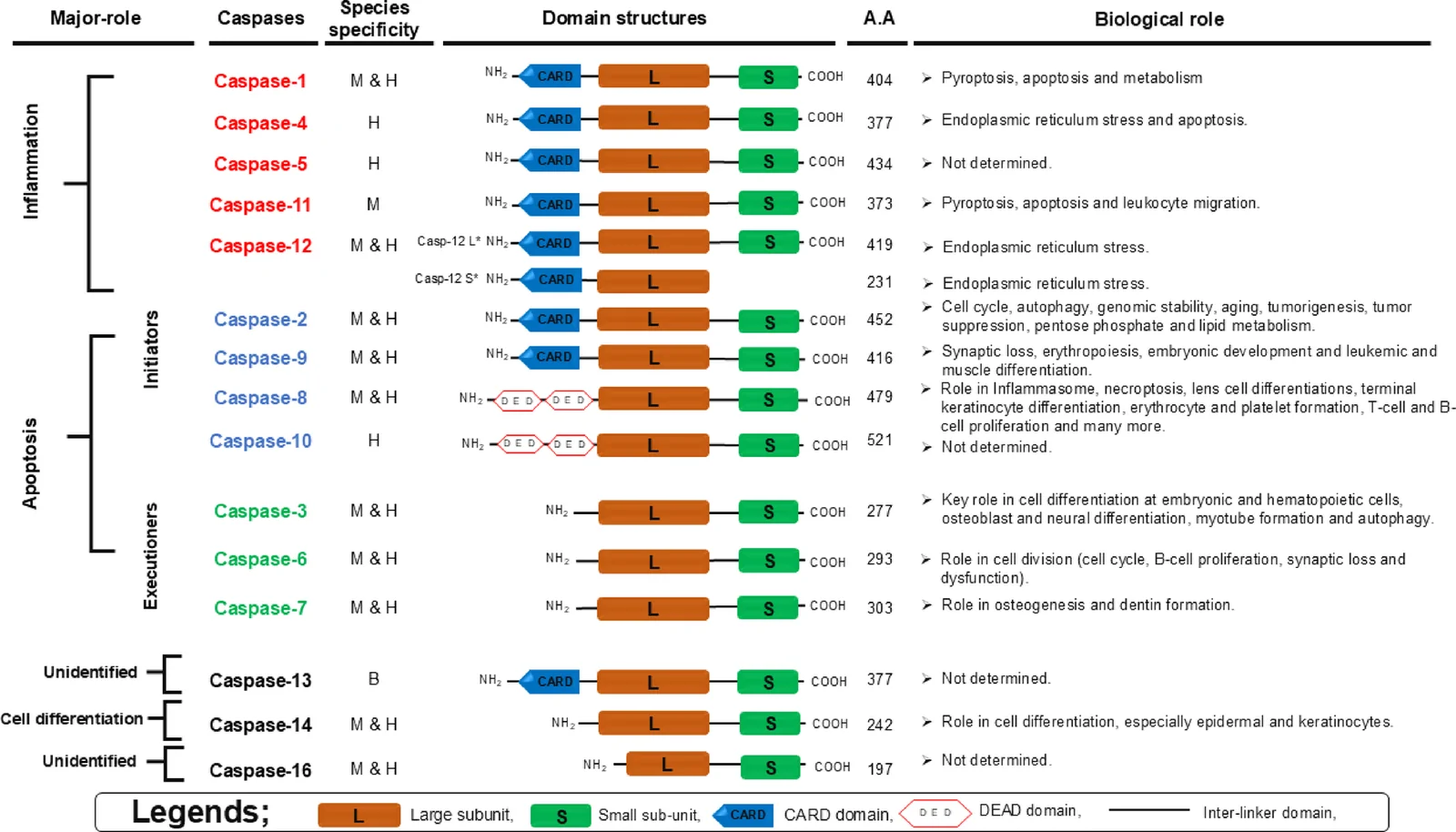
The figure illustrates the structural architecture and classification of mammalian caspases based on their biological role and domain composition. For example, inflammatory caspases, involving caspase-1, -4, -5, -11 and -12, are actively characterized by the presence of an N-terminal caspase recruitment domain (CARD), which mediates inflammatory responses. Similarly, apoptotic caspases encompass initiator caspases (caspase-2, -8, -9 and -10) that contain either a CARD or a death effector domain (DED), enabling them to recruit to activation complexes such as the apoptosome or the death-inducing signalling complex (DISC). In contrast, executioner caspases, including caspase-3, -6 and -7 possess short prodomains and play a key role in the downstream of initiator caspases to mediate proteolytic cleavage of cellular substrates during the process of apoptosis. In a similar manner, the catalytic region of caspases is mainly composed of a large (L) and small (S) subunit, which together form the active heterotetrameric enzyme following proteolytic maturation. The figure is assembled and modified in PowerPoint and derived from (Shalini et al. 2015; Fang and Peng 2022)

Caspases are cysteine-aspartate proteases that have diverse functions in living organisms. For example, caspases like caspase-1, have functions related to inflammation and innate immunity, while other caspases like caspase-3 and caspase-7, contributes in apoptosis (Sahoo et al. 2023). Caspase-10 takes part in the apoptotic signaling in humans while caspase-12 plays a role in ER stress response. The caspase-14 enzyme plays a role in skin differentiation (Eckhart et al. 2008). Through these distinct roles, caspases contribute in maintaining cellular homeostasis and coordinating appropriate responses to cellular stress and other pathogenic challenges (Kumar 2007). All caspases have a similar basic structure and function in the same general way, but activated differently and targeting different proteins for cleavage Although the fold and catalytic activity of caspases are highly conserved, their activation mechanism and substrate specificity are different (Stegh and Peter 2001). In the human body, caspases perform a unique role in apoptosis and other biological processes related to programmed cell death (Eckhart et al. 2008). Caspases are classified based on their structure, domain organization and biological function, including their roles in apoptosis and inflammatory signaling (Fig. 1).

### Inflammatory caspases

Inflammatory caspases include caspase-1, -4, and -5 in humans, and caspase-1 and caspase-11 in mice, where caspase-11 is the functional ortholog of human caspase-4 and -5. (Martinon and Tschopp 2007). Caspase-12 is also expressed in mice, although its function is more limited than in humans. The caspases are primarily expressed in vertebrates and they have an involvement in the innate immunity (Sakamaki and Satou 2009). Caspases become activated through protein multiprotein complexes called inflammasome which facilitates activation of particular inflammatory caspases (Shinkai et al. 2005) In the caspase family, caspase-1 is the one that is highly important since its role involves the cleavage of inactive cytokines IL-1β and IL-18. Upon activation, caspase-1 results to an apoptotic process called pyroptosis. (Sun and Scott 2016).

### Apoptotic caspases

#### Initiator caspases

This class of cysteine proteases encompasses caspases-2, 8, 9, and -10. Among them, Caspase-2, 8 and 9 are thoroughly present both in human as well as mice, while caspase-10 unique to human only (Sahoo et al. 2023). Initiator caspases are produced as inactive precursors and possess elongated pro-domains such as the death effector domain (DED) or the caspase recruitment domain (CARD) that enable their interaction with certain signaling complexes (Salvesen and Dixit 1999). The pro-domains of initiator caspases undergo auto-processing following dimerization *via* the involvement of oligomeric signaling complexes (Salvesen 2002; Bao and Shi 2007). Additionally, they commence the execution phase of apoptosis upon receiving particular death signals. All these caspases are responsible for the initiation of apoptosis, thereby acting as the top precursor of caspase cascade signaling pathway (Chen and Wang 2002).

#### Executioner caspases

This class of caspases encompasses caspases-3, -6 and -7 and are entirely activated by initiator caspases (Slee et al. 2001). Executioner caspases are also called effector caspases. Upon activation, the role of executioner caspases is synchronized to diminish essential structural proteins and stimulate other enzymes. resulting in their morphological, structural, and biochemical alteration associated with apoptosis (Nano et al. 2023). Both intrinsic and extrinsic apoptotic TNF-associated apoptosis-induced ligand (TRAIL) signaling pathways are triggered by initiator caspases, which in turn enhance the activation of other caspases, such as executioner caspases, leading to the cleavage of cellular components and ultimately causing apoptosis. More interestingly, initiator caspases have been thoroughly identified in humans as well as mice (Horuz et al. 2013; Hojo-Souza et al. 2015).

#### Other caspases

The origin and role of certain caspases, such as caspase-13, -14 and -16, are still under debate. However, among them, caspase-14 is a unique member of the caspase-1 family due to its highly tissue-specific expression pattern, being predominantly restricted to mammalian confining epithelia (Lippens et al. 2004). It is mainly expressed in the differentiating epidermal layers and hair follicles, where its distribution appears highly conserved among other species (Alibardi et al. 2004, 2005). In contrast, the existence and functional relevance of caspase-16 remain controversial, although it was initially identified as a caspase-14-like protease in several mammals (Eckhart et al. 2025). However, the structure and function of its human ortholog are still under consideration, and some studies, for example, Eskhart et al. (2008) and Sakamaki and Satou (2009) propose that it is a pseudogene.

Despite its inclusion in recent reviews of the human caspase repertoire, there is no definitive evidence confirming the expression of an appropriate and effective caspase-16 protease in humans. Furthermore, caspase-13 represents another controversial member of the caspase family. Although it was first investigated in 1998 by Humke et al. (1998), the subsequent finding failed to detect its expression in human tissues, contradicting earlier findings based on Northern blot analysis. However, later studies further demonstrated that caspase-13 does not constitute a genuine member of the human caspase-1 family, but exists in bovines (Taylor et al. 2000; Koenig et al. 2001).

### Structural and functional insights of caspase-1

Caspase-1 also known as interleukin-1 converting enzyme (ICE) is one of the key enzyme belong to a group of enzymes called aspartate-specific cysteine proteases (Vande Walle and Lamkanfi 2011)This was thought to be a unique proteolytic characteristic, holding an evolutionarily conserved enzyme that cleaves precursor proteins into their active forms i.e. the maturation of interleukin-1β and interleukin-18 followed by the pyroptosis inducer gasdermin D (GSDMD) into active mature peptides (Sollberger et al. 2014; McNair et al. 2018). Upon caspase-1 activation *via* inflammasomes and/or apoptosis, it progressively increasing the inflammation response in the cells (Kumaresan et al. 2016). Although some studies have claimed that proIL-1β and proIL-18 can also be activated in the absence of caspase-1 by some other proteases, especially in neutrophil cells (Guma et al. 2009; Joosten et al. 2009)Therefore, before discussing the maturation, caspase-1 is present in the inactive form known as ‘zymogen’ and can be activated by recruiting to a proper and well-known molecular platform termed the inflammasome (Elliott et al. 2009).

Inflammasomes that lead to the activation of caspase-1 are primarily made up of pattern-recognition receptors (PRRs) that are part of the NOD-like receptor (NLR) family. This includes NLRP1, NLRP3, NLRP6, NLRP7 and NLRC4. Apart from NLRs, there are also other types of sensors such as AIM2 that detect DNA and RIG-I that detect viral RNA (Hauenstein et al. 2015). These inflammasomes are primarily composed of several key domains, including a leucine-rich repeat domain, essential for sensing pathogen-associated molecular patterns (PAMPs) and/or damage-associated molecular patterns (DAMPs), a nucleotide-binding domain (required for oligomerization) and a caspase-1 activation and recruitment (CARD) domain, as well as a PYD domain for recruitment to caspase-1 (Chopra et al. 2024). However, among all above listed inflammasomes, NLRP3 is noted to be the most important inflammasome, especially in the inflammatory pathway, and can be activated by different stimuli like PAMPs and DAMPs (Jin and Flavell 2010; Carriere et al. 2021).

A single pro-caspase-1 (p45) is responsible for the creating of two sub-units i.e. p20 and p10, respectively (Romanowski et al. 2004). The activation of pro-caspase-1 can be achieved, thereby cleaving it to produce 20 kDa (p20) and 10 kDa (p10) subunits, respectively (Thornberry 1997; Mariathasan et al. 2004). Active caspase-1 consist of a catalytic domain as well as an active site, which forming catalytic dyad consisting of C285 and H237, respectively (Clark 2016). Similarly, during the cleavage of pro-caspase-1 a 119-residue pro-peptide and an 18-residue sequences are removed seperates the mature enzyme’s large (p20; residues 120–298) and small (p10; residues 317–404) subunits (Wilson et al. 1994). On the same way, auto-proteolytic cleavage at three aspartic acid residues are the main sites of this (D119, D297 and D316), resulting in the release of the pro-domains, i.e., caspase recruitment domain (CARD), caspase docking loop (CDL) and interdomain linker (IDL) and serving as a crucial step in the activation of caspase-1 (Lu et al. 2016).

Additionally, the active/mature caspase-1 consists of two heterodimers of p20 and p10. The catalytic domain has an active site that goes across both subunits (Huang et al. 2009); Boucher et al. 2018). Active caspase-1 actively interact with other protein such as apoptosis-associated speck-like protein containing a (ASC) and Nod-Like Receptor (NLR) proteins, which are caspase activation recruitment domain (CARD) proteins forming inflammasomes (Huang et al. 2009). Similarly, Caspase Activation and Recruitment Domain (CARD) that interacts with other proteins, such as Apoptosis-Associated Speck-like Protein Containing a CARD (ASC) and Nod-Like Receptor (NLR) Family CARD Domain-Containing Protein 4 (NLRC4) (Newton and Dixit 2003; Jorgensen and Miao 2015).

### NLRP3: occurrence, activation, and role in inflammation

**Fig. 2:**
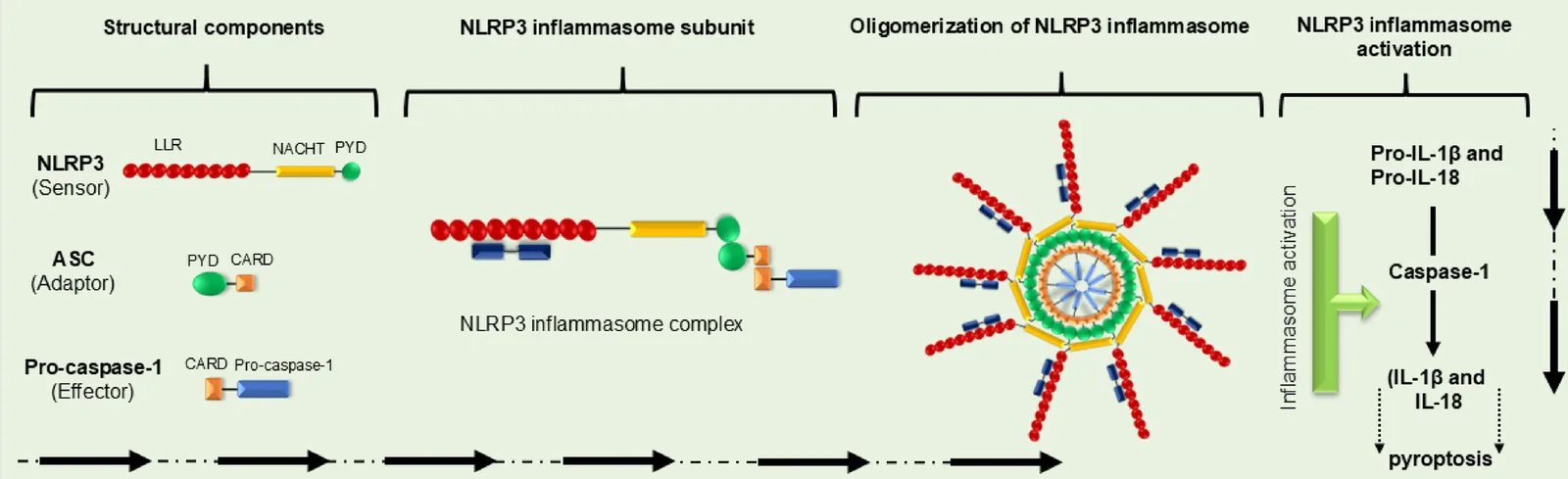
Structural organization, assembly, oligomerization and activation. Here the NLRP3 inflammasome is a multiprotein complex composed of three main components: the sensor protein NLRP, the adaptor ASC (apoptosis-associated speck-like protein containing caspase recruitment domain and pro-caspse-1. Similarly, NLRP3 in its inactive form consisted of different components characterized by the presence of leucine-rich repeat (LLR), followed by NACHT and pyrin (PYD) domains. Upon sensing cellular stress signals in the form of PAMS and DPAMS, NLRP3 undergoes ATP-dependent conformational changes and oligomerization. The activated NLRP3 recruits ASC through PYD-mediated interactions and ASC subsequently recruits pro-caspase-1 through caspase recruitment domain (CARD)-mediated interactions. This multiprotein assembly promotes proximity-induced activation of caspase-1, which further catalyzes the maturation of proinflammatory cytokines pro-IL-1β and pro-IL-18 into their active forms IL-1β and IL-18, respectively. The release of these cytokines triggers downstream inflammatory signaling and contributes to innate immune defense response. The figure was designed in the Microsoft PowerPoint 2019

Inflammasome are the class of cytosolic protein complexes that are formed in order to mediate host immunological responses to microbial infection and cellular stresses, infections and/or injuries (Yi 2020). Inflammasome like NLRP3 The NLRP3 inflammasome is a multi-protein complex comprising the apoptosis-associated speck-like protein (ASC), which contains a caspase recruitment domain (CARD) and a pro-caspase-1 domain. NLRP3 inflammasome is present in immune cells like neutrophils, macrophages, epithelial cells and also in non-immune cells (Jin and Flavell 2010). Inflammasome activation is initiated by pathogen-associated molecular patterns (PAMPs) and damage-associated molecular patterns (DAMPs), which are recognized through pattern-recognition receptors and trigger the assembly of inflammasome complexes, including ‌NLRP3 (Shi et al. 2016). Upon activation, the NLRP3 inflammasome recruits the adoptor protein apoptosis-associated speck-like protein containing a CARD (ASC), facilitating the recruitment and autocatalytic activation of caspase-1 (Fig. 2). Interestingly, similar mechanisms have also been described in parasitic pathogens, such as *Schistosoma mansoni*, which activate ASC-NLRP3 inflammasome axis, resulting caspase-1 activation and subsequent maturation of cytokines i.e. IL-1β and IL-18, thereby promoting inflammatory and innate immune responses (Sanches et al. 2020). In addition, emerging evidences indicates that caspase-4 contributes to the expression and functional activation of the NLRP3 inflammasome in THP-1 cells and human keratinocytes, highlighting the complex interplay between inflammatory caspases and regulating inflammasome signalling (Garrido et al. 2019).

The activation of the NLRP3 inflammasome is widely recognized as a two-step mechanism comprising a priming signal followed by an activation signal (Sutterwala et al. 2014). In the very first stage (known as priming), the transcriptional upregulation of NLRP3 and the inactive cytokine precursors, including pro-IL-1β and IL-18 (Swanson et al. 2019). This process is typically initiated by the activation of pattern-recognition receptors (PRRs), particularly, toll-like receptors (TLRs), following the recognition of pathogen-which detect damage-associated molecular pattern (DAMPs) and/or pathogens-associated molecular pattern (PAMPs), which activate the NF-κB signalling pathway (Sutterwala et al. 2014). Similarly, a well-characterized example of a priming stimulus lipopolysaccharide (LPS), a major structural component of the outer membrane of gram-negative bacteria and is recognized by TLR4. In contrast, endogenous danger signals such as uric acid crystal act as DAMPs and contribute to inflammasome activation in response to cellular stress and/or tissues injuries (Martinon et al. 2006).

The‍‌‍‍‌‍‌‍‍‌ second tier of NLRP3 inflammasome activation is triggered by a wide range of cellular stress signals, including the efflux of potassium (K^+^) and chloride (Cl^−^) ions, the influx of calcium (Ca^2+^), accumulations of cytosolic nucleic acids, metabolic perturbations, lysosomal disruption, mitochondrial dysfunctions and the generation of reactive oxygen species (ROS) (Li et al. 2020). Although, these are the stimuli/signals are different in origin and structure, however, they converge on common cellular events that promote NLRP3 activation. Similarly, among the key regulatory proteins Never in Mitosis Gene A-related Kinase 7 (NEK7) and thioredoxin-interacting protein (TXNIP) actively facilitate NLRP3 activation by promoting conformational changes and oligomerization of the receptor‍‌‍‌‍‍‌ (Shen and Abe 2019). The activated NLRP3 subsequently undergoing oligomerization and recruits the adaptor protein apoptosis-associated speck-like protein containing a CARD (ASC) through and PYD-PYD) interactions. ASC then polymerizes into filamentous structures that assemble into a well-defined characteristic ASC speck, which serves a molecular platform for the recruitment of procaspase-1 *via* caspase recruitment domain CARD-CARD interactions, ultimately leading to the caspase-1 activation (Isazadeh et al. 2022).

**Fig. 3:**
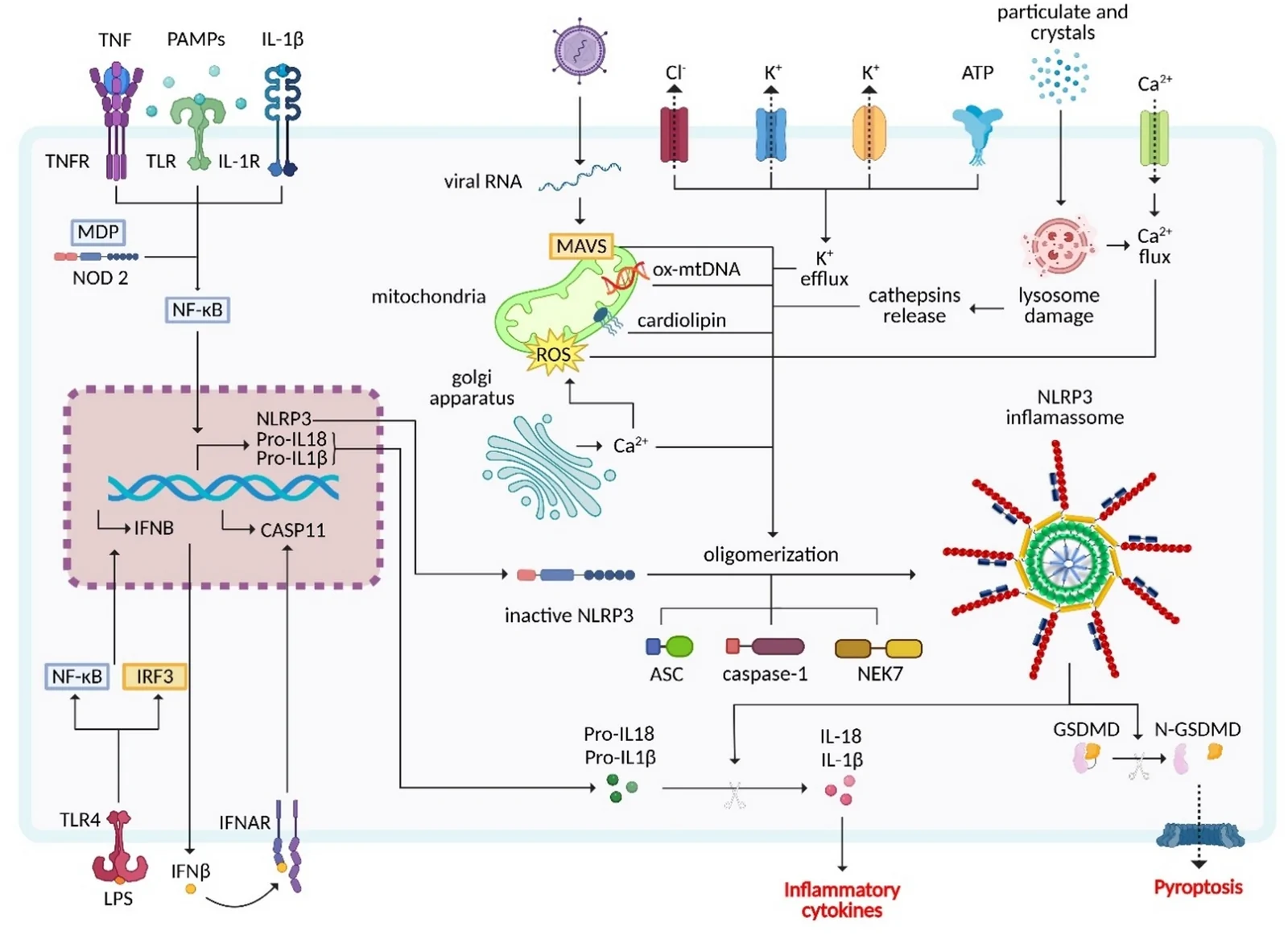
Schematic representation of the canonical and non-canonical signaling pathway involved in NLRP3 inflammasome activation that leads to the activation of caspase-1 and ultimately pyroptosis. The figure actively illustrates the priming, assembly, and activation mechanisms of the NLRP3 inflammasome triggered by diverse inflammatory stimuli, including TNF, followed by IL-1β, PAMPs, viral RNA, ATP, ions influxes (K^+^, Cl^−^ and Ca^2+^). Similarly, cellular contents comprise lysosomal damage, mitochondrial damage, and radicals in the form of ROS and particulate crystals. These signals activate NF-κβ/IRF3-mediated transcription of NLRP3, pro-IL-1β, and pro-IL-18 and caspase-11, while mitochondrial ROS, oxidized mtDNA, MAVS, cardiolipin, cathepsin release, and NEK3 promote NLRP3 oligomerization. The assembled inflammasome recruits ASC and pro-caspase-1, leading to caspase-1 activation and, therefore, the maturation of IL-1β and IL-18 takes place, and cleavage of GSDMD into N-GSDMD and induction of pyroptosis cell death, accompanied by inflammatory cytokine release. The figure was adopted from (Molla et al. 2020) using BioRender (https://www.biorender.com/) and Microsoft PowerPoint 2019

Following activation, NLRP3 under oligomerization, exposing its N-terminal pyrin domains (PYDs), which recruit the adaptor protein apoptosis-associated speck-like protein containing CARD (ASC) through homotypic PYD-PYD interactions (Oroz et al. 2016). ASC subsequently polymerizes into filamentous structures that assemble into large perinuclear ASC speck, providing a molecular platform for the recruitment of procaspase-1 *via* caspase-1 recruitment of domain (CARD-CARD) interactions (Hauenstein et al. 2015). Similarly, the close proximity of procaspase-1 molecules with the inflammasome promotes their autocatalytic cleavage, generating enzymatically active caspase-1. thus, it is the main enzyme that brings about the whole inflammatory response (Kesavardhana and Kanneganti 2017). The activated caspase-1 then proteolytically processes the inactive cytokines pro-inflammatory cytokines (pro-IL-1β and pro-IL-18) into their mature, biologically active forms and cleave gasdermin D (GSDMD). The released N-terminal fragment of GSDMD oligomerizes and inserts into the plasma membrane to form transmembrane pores, actively facilitating the release and secretion of IL-1β and IL-18 and ultimately triggering pyroptosis, a highly inflammatory form of programmed cell death characterized by membrane rupture and release of intracellular inflammatory mediators (IL-1β and IL-18) (Hu 2025) (Fig. 3).

### Role of caspase-1 inhibitors in inflammation

Caspase-1 inhibitors play a crucial role in regulating excessive inflammatory reactions, thereby preventing the activation of pro-inflammatory cytokines, including IL-1β and IL-18 (Molla et al. 2020). These inhibitors are important in the treatment of chronic inflammatory illnesses like gout, rheumatoid arthritis, and inflammatory bowel problems, where persistent inflammation causes substantial morbidity and tissue damage (Lamkanfi et al. 2007; Sun and Scott 2016). Similarly, these caspase-1 inhibitors can decrease the inflammation, thereby enhancing the quality of life and relieving symptoms in autoimmune disorders like multiple sclerosis and systemic erythematosus, where aberrant immune system activation causes substantial harm and destruction in the body (Denes et al. 2012).

Likewise, caspase-1 inhibitors could prove useful as therapeutic agents, particularly in neuroinflammatory conditions such as Parkinson’s and Alzheimer’s, where inflammation plays a significant role in disease progression (Flores et al. 2022). Interestingly, by decreasing caspase-1 expression and lowering neuroinflammation, these inhibitors may be able to prevent the worsening of these debilitating disorders (Gao et al. 2020). Additionally, caspase-1 inhibitors provide a novel way to lower these inflammatory processes and enhance cardiovascular health in cardiovascular disorders, when inflammation leads to atherosclerosis and ultimately heart failure (Dhani et al. 2021).

On the other hand, several risks are directly and indirectly associated with caspase-1 inhibition (Randle et al. 2001). For example, with the complete/permanent inhibition of caspase-1 activities, the immune system may be less able to respond and combat infections caused by inflammation, a crucial factor in the immune defense system (Sollberger et al. 2014). This might be more vulnerable to infections by bacteria, viruses, fungi, and other infectious microbes (Vande Walle and Lamkanfi 2011). However, prolonged usage of caspase-1 inhibitors may also compromise serious surveillance that may lead to the emergence of malignancies (cancers) to develop, accordingly. Caspase-1 inhibitors can only be used effectively and safely once the risks are weighed against their potential therapeutic applications (Randle et al. 2001; Dhani et al. 2021).

Maximizing the safety and efficacy of caspase-1 inhibitors is of key interest and urgency, hence several approaches have been considered to identify potential inhibitors from different sources.

### Caspase-1 inhibitors

#### In silico

The term “in silico inhibitors” refers to drug molecules designed, screened, and/or optimized using different computer simulations rather than traditional laboratory experiments. This computational technique applies different approaches, such as molecular docking, where researchers simulate interactions between potential drugs and target proteins/enzymes, in order to identify compounds that have the capability to inhibit their activity. This is an easy, reliable, quicker, and cheaper method than experimental screening, which is time-consuming, expensive, and requires a well-designed setup. Several researchers have used in silico alone and in silico-combined with their experimental findings (in vitro and in vivo) to search for a possible drug candidate for caspase-1.

Through a comprehensive overview of in silico-identified and experimentally validated inhibitors of caspase-1, as listed in Table 1. Among them, Gonzalez-Cofrade and his colleagues (González-Cofrade et al. 2023) presented a group of bioactive compounds, including colchicine, dexamethasone, raloxifene, and methylprednisolone derived from *Colchicum autumnale*, which were identified through in silico screening as potential caspase-1 inhibitors (Caruso et al. 2022). Among the tested compounds, triterpenes 6 and 14 were among the most potent caspase-1 activity inhibitors, with IL-β secreation (IC_50_ = 1.15 µM and 0.19µM, respectively), and pyroptosis (IC_50_ = 2.21µM and 0.13µM, respectively).

Similarly, quinone methide triterpenoids such as hydroxytingenone, tingenone, and pristimerin isolated from *Maytenus octogona*, demonstrated inhibitory potential against caspase-1 through defined in silico and in vitro approaches (Caruso et al. 2023). Furthermore, other related compounds reported by Gonzalez-Cofrade and his colleagues (González-Cofrade et al. 2023), including nor-triterpene (6) and nor-triterpenequinone (14) isolated from *Maytenus retusa*, exhibited dual targeting of NLRP3 and caspase-1. Authors confirmed their selective inhibition of NLRP3 inflammasome, both canonical and non-canonical activation chemical pathways, without any interference in the AIM2 and/or Nod-Like Receptor Family CARD Domain-Containing Protein 4 (NLRC4) signalling pathways, respectively.

Furthermore, another study conducted by Yu et al. (2023) found certain phytochemicals (eleutheroside A, liriodenrin, epicatechin, 2-methoxy-4vinylphenol, catechin, androsin, coumaroylyramine and catechol) derived from *Sargentodoxa cuneata*. These compounds were identified through in silico studies mainly targeting the TLR4/NF-κB/NLRP3 signalling axis, indicating upstream regulation of inflammasome activation. Similarly, the in vivo extract significantly reduced disease efficacy in terms of colon injury, colon shortening, and spleen enlargement in treated rats with lowered colonic levels of TNF-α, IL-1β, IL-6, and IL-17; and downregulation of TLR4, NF-κβ p65, NLRP3, and caspase-1 mRNA expression.

Several synthetic small molecules were also reported, including 2-(2S,8S,11S)-11-([1,1’-Bi-phenyl]-4-ylmethyl)-8-benzyl-3,6,9,12-tetraoxo-1,4,7,10-tetraazacyclododecan-2-yl) acetic acid (Olujinmi et al. 2025), aspartyl acyloxyalkyl ketones and aspartyl amidooxyalkyl ketones (Galatsis et al. 2010) and triaminopyrimidine (Grice et al. 2024), all predicted as caspase-1 inhibitors. Moreover, Galatsis et al. (2010) further confirmed the most efficient and potent inhibitor, 4i, which exhibited a Ki of 0.5 µM. It was concluded that this compound series enhances the understanding of the hydrophobic and prime-side binding requirements of caspase-1.

In the same way, Fournier et al. (2018) predicted uracil 20 compound, a potent caspase-1 inhibitor possessing (IC_50_ = 38 nM in the THP-1 cells), specially developed for topical use in inflammatory acne (Fournier et al. 2018). In addition, Patel et al. (2018b) investigated 2, 4-diaminopyrimidine derivatives, among all six compounds, named 6 m, 6n, 6o, 6p, 6q, and 6r, which demonstrated potent enzymatic inhibition, with IC_50_ ranging from 0.022 to 0.079 µM, and showed strong cellular activity at sub-micromolar concentrations. Furthermore, molecular docking studies further revealed key interactions supporting their selectivity for caspase-1.

The chemical universe is quite diverse, and it’s believed that it encompasses up to 10^6^ small molecules. To have easy access, accelerate drug discovery and biological research, diverse chemical libraries were created for public use (Bohacek et al. 1996; Kuan et al. 2023). With the same context, database-driven virtual screening further identified several promising and interesting candidates as caspase-1 inhibitors. For example, from the ZINC database, compounds such as belnacasan (VX-765) (Li et al. 2022), ZINC00885612, ZINC72003647, BTBO4175, and BTB04410 (Patel et al. 2018a); and ZINC724667, ZINC9908119, and ZINC09933770 (Kumi et al. 2020), exhibited strong binding affinities towards caspase-1.

Similarly, another commercial compound (NSC697923) was also identified through in silico screening, which possesses caspase-1 inhibitory potential (Cao et al. 2022). Likewise, compounds retrieved from ChEMBL, including linagliptin, icariin, and rolipram, interestingly exhibited dual inhibition of caspase-1 and TNF-α, indicating broader anti-inflammatory potential (Speck-Planche et al. 2021). Additionally, blumeatin and luteolin from PubChem were predicted as potential caspase-1 inhibitors (Pratama et al. 2022), while colchicine was also independently validated via virtual screening targeting caspase-1 and GSTO-1 (glutathione-S-transferase omega-1) (Satrijo et al. 2025). Notably, curcumin, sourced from Sigma-Aldrich, demonstrated multi-target inhibition involving TREM-1, DAP12, NLRP3, caspase-1, and IL-1β, supported by both in vitro and in silico studies, underscoring its pleiotropic inflammatory effects (Nguyen et al. 2022).

The use of in silico-based approaches involving different methodologies, especially molecular docking, virtual screening, structure-based drug design, network pharmacology, QSAR, and molecular dynamics (MD) simulations (De Vita et al. 2023). These approaches have substantially broadened the landscape of caspase-1 inhibitor discovery, thereby enabling the rapid identification of structurally diverse compounds. Interestingly, these compounds are derived from extractions, phytochemical libraries, synthetic molecules, and online publicly available libraries/databases (Ezzat et al. 2019). The said computational approaches, complemented by in vitro and in vivo validation, not only confirm binding affinities and inhibitory potential but also provide mechanistic insights into ligand-target interactions at the molecular level (Fang 2012).

Furthermore, it’s quite amazing that the integration of in silico, followed by in vitro and in some cases in vivo approaches, enhances the predictive accuracy, efficiency, and translational relevance of these findings (Stanimirovic et al. 2015). Similarly, computational screening significantly reduces time, efforts, equipment, and costs compared to traditional drug discovery pipelines, while experimental validation ensures biological reliability (Lin et al. 2020). Collectively, this synergistic approach accelerates the identification and optimization of novel anti-inflammatory agents with improved specificity, reduced toxicities, and therapeutic applicability in inflammatory-related disorders.

**Table 1:** Summary of different inhibitors derived from different sources for the inhibition if caspase-1 (direct inhibitors) and other components in the signaling pathway (in direct caspase-1 inhibitors), involving in silico and other approaches

Compounds	Sources of compounds	Targets	Mechanism classification	Method used	References
Colchicine, dexamethasone, raloxifene, and methylprednisolone	Phytochemicals isolated from *Colchicum autumnale*	Caspase-1	Direct caspase-1 inhibitor	In silico	Caruso et al. (2022)
Hydroxytingenone, tingenone, and pristimerin	Phytochemicals isolated from the aerial parts of *Maytenus octogona*	Caspase-1	Direct caspase-1 inhibitor	In silico and in vitro	Caruso et al. (2023)
Triterpenes 6 (nor-triterpene) and nor-triterpenequinone (14)	Phytochemicals: isolated from the aerial parts of *Maytenus retusa*	NLRP3/Caspase-1	Indirect (NLRP3-dependent)	In silico and in vitro	González-Cofrade et al. (2023)
Eleutheroside A, liriodenrin, epicatechin, 2-methoxy-4vinylphenol, catechin, androsin, coumaroylyramine and catechol	Phytochemicals isolated from *Sargentodoxa cuneata*	TLR4/NF-κβ/NLRF3	Indirect caspase-1 inhibitor	In silico	Yu et al. (2023)
2-(2S,8S,11S)-11-([1,1’-Bi-phenyl]-4-ylmethyl)-8-benzyl-3,6,9,12-tetraoxo-1,4,7,10-tetraazacyclododecan-2-yl) acetic acid	Synthetic	Caspase-1	Direct caspase-1 inhibitor	….	Olujinmi et al. (2025)
Aspartyl acyloxyalkyl ketones and aspartyl amidooxyalkyl ketones	Synthetic	Caspase-1	Direct caspase-1 inhibitor	….	Galatsis et al. (2010)
NSC697923	Commercial	Caspase-1	Direct caspase-1 inhibitor	In silico	Cao et al. (2022)
Uracil 20	Synthetic	Caspase-1	Direct caspase-1 inhibitor	…	Fournier et al. (2018)
Triaminopyrimdine	Synthetic	Caspase-1	Direct caspase-1 inhibitor	….	Grice et al. (2024)
Belnacasan	Zinc database	Caspase-1	Direct caspase-1 inhibitor	In silico	Li et al. (2022)
ZINC00885612, ZINC72003647, BTBO4175 and BTB04410	Zinc database	Caspase-1	Direct caspase-1 inhibitor	In silico	Patel et al. (2018a)
Linagliptin, icariin, and rolipram	ChEMBL	Caspase-1/TNF-alpha	Indirect caspase-1 inhibitor	In silico	Speck-Planche et al. (2021)
ZINC724667, ZINC9908119 and ZINC09933770	Zinc database	Caspase-1	Direct caspase-1 inhibitor	In silico	Kumi et al. (2020)
Blumeatin and luteolin	Pubchem	Caspase-1	Direct caspase-1 inhibitor	….	Pratama et al. (2022)
Curcunmin	Sigma-Aldrich	TREM-1/DAP12/NLRP3/Caspase-1/IL-1β	Indirect caspase-1 inhibitor	In vitro and in silico	Nguyen et al. (2022)
2, 4-diaminopyrimidine derivatives	Synthetic	Caspase-1	Direct caspase-1 inhibitor	In vitro and in silico	Patel et al. (2018b)
Colchicine	Pubchem	Caspase-1/GSTO-1	Indirect caspase-1 inhibitor	In silico	Satrijo et al. (﻿^2025^)

Where “…” indicate not reported, interleukin 1beta (IL-1β), interleukin 18 (IL-18), nod-like receptors family pyrin domain containing 3 (NLRP3), glutathione-S-transferase omega-1 (GSTO-1), triggering receptor expressed on myeloid cells-1 (TREM-1) and DNAX-activation protein of 12 kDa (DAP12)

### In vitro and in vivo

Previously, several researchers have tried their best to find a possible inhibitor for caspase-1; enlisted in Table 2. Furthermore, several research groups have also targeted the NLRP3 inflammasome and related components in the chemical pathway, along with their sources, molecular targets, experimental validations, cytotoxicities, and IC_50_ values, respectively.

Liu et al. (2023) investigated a multi-component formulation (JZ-1) containing berberine, astilbin, paeonol, neoastilbin, paeoniflorin and dictamnine. The authors noted that JZ-1 significantly inhibited viral replication (IC_50_ = 1.709 mg/mL) and effectively reduced caspase-1-dependent pyroptosis. Similarly, Ahmad et al. (2013) carried out the anti-metastatic and immunomodulatory potential of thymoquinone (2-isopropyl-5-methylbenzo-1,4-quinone), from *Nigella sativa* (black seed) in mouse (B16F10 and human (A375) melanoma cell lines, respectively. Their findings revealed that NLRP3 inflammasome persistently activated in the melanoma cells, which contributes to inflammation and metastasis *via* caspase-1 activation and the secretion of key pro-inflammatory cytokines IL-1β and IL-18. Furthermore, Lopez-Tenorio et al. (2020) investigated morin (3, 5,7, 2′,4′-pentahydroxyflavne) and polyunsaturated fatty acids (PUFAs), which significantly reduced the expression of IL-18, caspase-1, peroxisome proliferator-activated receptor gamma (Pparγ) and sterol regulatory element-binding protein 1c (Srebp-1c), while upregulating peroxisome proliferator-activated receptor alpha (Ppar-α) and adiponectin expression levels. It was quite amazing that medicinal plants contain interesting phytochemicals that have been shown to exhibit inflammasome-inhibiting qualities (Tőzsér and Benkő 2016).

The immune modulation effect of polyphenols is well-documented and has been shown to affect immune cell populations, regulate the generation of cytokines and alter the expression of pro-inflammatory genes (John et al. 2011; Karasawa et al. 2011). For example, Rodrigues et al. (2021) reported the anti-inflammatory and metabolic potential of polyphenolic compounds (including ellagic acid derivatives like anthocyanins and flavanols) derived from the rigues *Plinia jaboticaba*, exhibited caspase-1 inhibition, followed by cytokines (TNF-α, TLR-4 and NF-κB) (Rodrigues et al. 2021). These effects were associated with suppression of the colonic inflammasome pathway, suggesting that *P. jaboticaba* phenolic compounds exert protective effects in obesity at least partly through inflammasome modulation, including the inhibition of caspase-1 activity.

Similarly, 6-gingerol from ginger presented anti-inflammatory effect, thereby inhibiting NOD-LRR and pyrin-domain containing protein 3, followed by caspase-1, IL-β and IL-18 in the brain tissues (Luo et al. 2021). In the same way, Xu et al. (2021) explored the neuroprotective role of Bakuchiol (BAK), from *Psoralea corylifolia*, which actively suppressed apoptosis-associated speck-like protein containing a CARD (ASC), NLRP3 and caspase-1 in a mouse model of cerebral ischemia-reperfusion (I/R) injury (Xu et al. 2021). Another study of Sebastian-Valverde et al. (2021) reported that malvidin-glucoside reduces inflammation, thereby inhibiting caspase-1 activities and increasing resistance to anxiety and depression brought on by stress conditions.

Flavonoids known to have antioxidant potentials that actively protect living cells/body against different complications (through inflammation), like cancer, heart disease and neurological disorders (Ramos 2007). The protective effect of these flavonoids may inhibit the NF-κB signalling pathway, resulting in the enhancement of apoptosis and decreasing cancer cell proliferation (Prasad et al. 2010). Several research groups have demonstrated that flavonoids such as baicalin (Zheng et al. 2021b), followed by chrysin (Huang et al. 2022) didymin (Zhang and RuXian 2022) quercetin and allopurinol (Wang et al. 2012). These all flavonoids consistently inhibited ASC, NLRP3, caspase-1 and actively down-regulated cytokines (IL-β, IL-18, IL-16 and TNF-α), respectively. Furthermore, quercetin also regulated the TXNIP-mediated inflammasome activation (Zhang et al. 2014). Similarly, flavonoids and diterpenoids from *Herba siegesbeckiae* actively inhibited the caspase-1 and pro-inflammatory cytokines, respectively (Wei et al. 2022).

Other polyphenols, including punicalagin (An et al. 2020), followed by procyanidins (Liu et al. 2022) and trilobatin (Zhang et al. 2022b), demonstrated multi-target inhibitions including NLRP3, followed by caspase-1, IL-β, IL-18 and Gasdermin D (GSDMD), indicating the suppression of pyroptosis. Furthermore, other compounds such as salvianolic acid A from *Salvia miltiorrhiza* (Ma et al. 2020), salidroside (Zheng et al. 2021a), caenosic acid (Song et al. 2018) and saxifragin (Cheon et al. 2015) primarily targeted NLRP3 and caspase-1, whereas chrysophanol (Kim et al. 2010) and naringin (Zeng et al. 2014). The above-mentioned compounds modulated COX-2 and NF-κB chemical pathways, respectively. Flavonoids present in the daily foodstuffs like fruits and vegetables, have been shown to reduce the expression of various pro-inflammatory cytokines and chemokines, such as TNFα, IL-1β, IL-6, IL-8 and monocyte-chemoattractant protein-1 (MCP-1), in various cell types, including Jurkat T-cells, RAW macrophages and peripheral blood mononuclear cells. The anti-inflammatory cytokine IL-10 was released more frequently as a result of quercetin and catechins’ inhibitory effects on TNFα and IL-1β (Santangelo et al. 2007).

Different alkaloids, like neferine derived from lotus seed embryos, possess numerous biological and pharmacological actions, including anti-tumor, anti-inflammatory, anti-oxidative, anti-fibrosis and anti-arrhythmic properties (Poornima et al. 2014). Because of its anti-inflammatory and anti-oxidative properties as well as its suppression of cytokines, neferine has demonstrated therapeutic efficacy in several conditions (Kadioglu et al. 2017; Marthandam Asokan et al. 2018). Similarly, alkaloids from *Catha edulis* (cathinone, cathine and norephedrine) showed potential caspase-1 inhibition with defined cytotoxic concentrations (Dimba et al. 2004), while hentriacontane from *Oldenlandia diffusa* (Kim et al. 2011) and total saponins from *Dioscorea collettii* (Wang et al. 2020) exhibited minimal or no cytotoxicity, indicating safer profiles. Moreover, other alkaloids like berberine were presented by Jin et al. (2017) and rhubarb anthraquinones by Zheng et al. (2024) also modulated certain upstream regulators such as TLR4 and NF-κB. It’s quite obvious that alkaloids have antioxidant and anti-inflammatory potentials. For example, sinomenine, an alkaloid isolated from *Sinomenium acutum* with a variety of pharmacological characteristics, such as anti-inflammatory, immunomodulatory, and anti-tumor effects (Li et al. 2023).

Likewise, potent synthetic inhibitors such as VX-765 and VRT-043198 demonstrated strong caspase-1 inhibition at nanomolar levels (3.96 and 9.91 nM) (Flores et al. 2020). Comparably, some traditional formulations, including Mahuang Fuzi Xixin (Ding et al. 2024) and Qiji Shujian granules (QJG), presented by (Huan et al. 2023), showed dose-dependent inhibition of the key regulators such as NLRP3/caspase-1/GSDMD-mediated pyroptosis pathway, supported by in vitro, in vivo, and in silico evidence. Furthermore, the NLRP3 overexpression significantly reduced cell viability, increased ROS and MDA production, and thereby enhanced NLRP3, GSDMD-N, caspase-1, and IL-1β; these pathological effects were markedly reversed by QJG treatment.

Furthermore, Liu et al. (2025) found that cordycepin significantly reduced reactive oxygen species (ROS) levels and xanthine oxidase (XO) activity, suppressed the expression of NLRP3/GSDMD, cleaved pro-caspase-1, and reduced IL-1β and IL-18. Other compounds, such as astragaloside ⅠⅤ (Xiao et al. 2021), followed by phenetheyl isothiocyanate (Wang et al. 2025), β-stosterol (Zhang et al. 2023), rosmarinic acid (Jeong et al. 2011), gastrodin (Sun et al. 2019) and isoliquiritigenin isolated from *Glycyrrhiza uralense* caused dose-dependent suppression of NLRP3 inflammasome-mediated LDH release and IL-1β production in CAPS model THP-1 cells expressing NLRP3-D303N and NLRP3-L353 mutations (Usui-Kawanishi et al. 2024). The above-mentioned diverse classes of compounds further confirmed inhibition of key inflammasome mediators, including TXNIP, ASC, NLRP3, caspase-1, GSDMD, and IL-1β.

From the current literature, the majority of inhibitors were validated through several experimental designs, i.e., in vitro and in vivo, with a few supported by in silico approaches. Almost all inhibitors in the studies consistently target ASC, NLRP3, caspase-1, IL-1β and IL18 axis. Similarly, cytotoxicity data were available, indicating generally favourable safety profiles, although IC_50_ values remained elusive for several compounds/extracts used. Due to the lack of IC_50_ in the studies loses its statistical rigor and comparative capacity, and the main flaw is the impossibility of defining the compounds’ true and exact potencies.

**Table 2:** Experimental and natural caspase-1 inhibitors

Inhibitors	Sources	Cytotoxicity	IC_50_	Targets	Assay	References	
Citric acid, d-(-)-quinic acid, berberine, caffeic acid, taxifolin, ferulic acid, citric acid, luteolin, and trigonelline	JZ-1	6.25 mg/mL	1.709 mg/mL	↓ Caspase-1	In vitro/in vivo	Liu et al. (2023)	
Thymoquinone (2-isopropyl-5-methylbenzo-1,4-quinone)	*Nigella sativa*	……	…….	↓ Caspase-1	In vitro/in vivo	Ahmad et al. (2013)	
Morin (flavonol)		….	….	↓ Caspase-1, IL-1β and IL-18	In vitro/in vivo	López-Tenorio et al. (2020)	
Ellagic acid and its derivatives (hydroxybenzoic acid), delphinidin-3-O-glucoside (anthocyanin), cyanidin 3-O-glucoside (anthocyanin), myricetin 3-O-rhamnoside (flavonol), quercetin 3-O-rhamnoside (flavonol)	Jaboticaba (*Plinia jaboticaba* (Vell.) Berg)	….	….	↓ Caspase-1, IL-1β and IL-18	In vitro/in vivo	Rodrigues et al. (2021)	
6-Gingerol	Ginger	….	….	↓ NLRP3, caspase-1, IL-1β and IL-18 (brain tissue)	In vitro/in vivo	Luo et al. (2021)	
Malvidin-glucoside	….	No	0.94µM	↓NLRC4/AIM2 and caspase-1	In vivo	Sebastian-Valverde et al. (2021)	
Bakuchiol (phenolic terpene)	*Psoralea corylifolia*	…	….	↓ ASC, NLRP3 and cleavedcaspase-1 (brain tissue)	In vitro/in vivo	Xu et al. (2021)	
Baicalin (flavone)	*Scutellaria baicalensis*	*….*	*….*	↓ LRP3, cleaved caspase-1, IL-1β and IL-18	In vitro/in vivo	Zheng et al. (2021b)	
Chrysin (flavone)		.	…	↓ ASC, NLRP3, caspase-1, IL-1β, IL-18, IL-6 and TNF-α	In vitro/in vivo	Huang et al. (2022)	
Flavonoids and diterpenes	*Herba Siegesbeckiae*	*…*	*…*	↓ Caspase-1, IL-1β, IL-18 and IL-6.	In vitro/in vivo	Wei et al. (2022)	
Didymin (flavanone)	*Citrus*	…	…	↓ ASC, NLRP3, caspase-1, IL-1β, IL-18 and TNF-α	In vitro/in vivo	Zhang and RuXian (2022)	
Quercetin (flavonol)	Commercial	…	…	↓ ASC, NLRP3 and caspase-1	In vitro/in vivo	Wang et al. (2012)	
Quercetin (flavonol)	Commercial	…	…	↓ TXNIP, NLRP3, caspase-1 and IL-1β	In vitro/in vivo	Zhang et al. (2014)	
Punicalagin	Pomegranate	…	…	↓ NLRP3, caspase-1, GSDMD and IL-1β	In vitro/in vivo	An et al. (2020)	
Salvianolic acid A	*Salvia miltiorrhiza* Bunge,	*…*	*…*	↓ NLRP3, caspase-1 and IL-1β	In vitro/in vivo	Ma et al. (2020)	
Salidroside	*Rhodiola rosea*	*…*	*…*	↓ TXNIP, pro-caspase-1, pro- IL-1β, IL-18, NLRP3 and caspase-1	In vitro/in vivo	Zheng et al. (2021a)	
Procyanidins (flavanol)	Commercial	…	…	↓ Pro-IL-1β, ASC, NLRP3, caspase-1 and IL-1	In vitro/in vivo	Liu et al. (2022)	
Trilobatin	*Lithocarpus polystachyus* Rehd,	…	…	↓ NLRP3, caspase-1, GSDMD, N-GSDMD, IL-1β and IL-18	In vitro/in vivo	Zhang et al. (2022b)	
Carnosic acid	Commercial	…	…	↓ NLRP3 and caspase-1	In vitro/in vivo	Song et al. (2018)	
Salidroside	*Rhodiola rosea*	*…*	*…*	↓ TXNIP, pro-caspase-1, caspase-1, pro-IL-lβ and IL-1β	In vitro/in vivo	Zheng et al. (2018)	
Saxifragin	*Saxifrage stolonifera*	*…*	*…*	↓ NLRP3, caspase-1	In vitro/in vivo	Cheon et al. (2015)	
Chrysophanol	Commercial	…	…	↓ COX-2, LPS, caspase-1 and NLRP3	In vitro/in vivo	Kim et al. (2010)	
Cathinone, cathine and norephedrin	Khat (*Catha edulis*)	8 × 10^−7^ M as compared to 2 × 10^−8^ M and 8 × 10^−8^ M	Yes	↓ Caspase-1	In vitro/in vivo	Dimba et al. (2004)	
Hentriacontane	*Oldenlandia diffusa*	*…*	*No*	↓ Caspas-1 and COX-2	In vitro/in vivo	Kim et al. (2011)	
VX-765 and VRT-043198	Commercial	3.69 and 9.91nM	…	↓ Caspase-1	In vitro/in vivo	Flores et al. (2020)	
Ephedrine, pseudoephedrine, higenamine, hypaconitine, aconine, benzoylhypaconine, aconitine and benzoylmesaconine.	Mahuang Fuzi Xixin	Doze-dependent		↓ NLRP3/caspase-1/GSDMD-N-mediated pyriptosis	In vitro/in vivo	Ding et al. (2024)	
Odoratin, bifendate and palbinone	Qiji Shujiang granule (drug)	10mM	…	↓ NLRP3, caspase-1, GSDMD and IL-1β	In vitro/in vivo/in silico	Huan et al. (2023)	
Sodium butyarte	Comercial	…	…	↓ NLRP3/caspase-1/GSDMD-N-mediated pyriptosis	In vitro/in vivo	Cui et al. (2025)	
Cordycepin	*Cordycepsmilitaris*	*…*	*…*	↓ NLRP3, caspase-1 and GSDMD	In silico/in vitro	Liu et al. (2025)	
Astragaloside IV	*Astragaloside membranaceus*	Yes	…	↓ NLRP3, caspase-1, GSDMD, GSDMD-N and IL-1β	In vitro/in vivo	Xiao et al. (2021)	
Phenethyl isothiocyanate	Cruciferous (vegetables)	…	…	↓ NLRP3, TXNIP, caspase-1 and IL-1β	In vitro/in vivo	Wang et al. (2025)	
β-sitosterol	Comercial	…	*…*	↓ NLRP3/ caspase-1/GSDMD-mediated pyroptosis	In vitro/in vivo	Zhang et al. (2023)	
Total saponin	*Dioscorea collettii*	No cytotoxicity	…	↓ Caspase-1 and NLRP3	In vitro	Wang et al. (2020)	
Astragaloside IV	*Astragaloside membranaceus*	*…*	…	↓ NLRP3, caspase-1 and GSDMD	In vitro/in vivo	Zhang et al. (2022a)	
Berberine	Coptidis rhizoma	…	*…*	↓ Caspase-1 and IL-1β	In vitro/in vivo	Jin et al. (2017)	
Free total rhubarb anthraquinone	Ruhbarb (peeled and dried roots)	…	…	↓ TLR4, NF-κβ, ASC, NLRP3, GSDMD and caspase-1	In vitro/in vivo	Zeng et al. (2024)	
Naringin	Comercial	…	…	↓ NF-κβ/COX-2-caspase-1	In vitro	Zeng et al. (2014)	
Rosmarinic acid	Densam-Eum (*Salvia mittiorriza*,* Sanatalum album and Amomum villosum*)	No cytotoxicity	*…*	↓ Caspase-1	In silico/in vitro	Jeong et al. (2011)	
Morin and 3, 5, 7, 2’, 4’-pentahydroxyflavone	Moraceae	No cytotoxicity	…	↓TXNIP/NLRP3/caspase-1/IL-1β	In vitro/in vivo	Zhou et al. (2021)	
Gastrodin	Component of traditional Chinese Medicine	…	…	↓ Caspase-1 and NLRP3	In vitro/in vivo	Sun et al. (2019)	
Isoliquiritigenin	*Glycyrrhiza uralensis*	*…*	*…*	↓ NLRP3	In vitro	Usui-Kawanishi et al. (﻿^2024^)	

Where “…” indicates not reported, ↓ inhibition/down-regulation, interleukin 1beta (IL-1β), interleukin 18 (IL-18), Nod-Like Receptors Family Pyrin Domain Containing 3 (NLRP3), caspase recruitment domain (CARD), thioredoxin-interacting protein (TXNIP), pyrin domain (PYD), Gasdermin D (GSDMD), lipopolysaccharide (LPS) and cyclooxygenase-2 (COX-2)

### Clinical and pre-clinical inhibitors for caspase-1

Several inhibitors from diverse origins have been used in pre-clinical and clinical trials targeting caspase-1, evaluated for inflammatory and neurodegenerative disorders (Table 3). A wide range of comprehensive approaches highlighting molecular targets, animal models and stages of pre-clinical and clinical developments. More interestingly, these inhibitors primarily target caspase-1-mediated inflammatory signaling, emphasizing the critical role of inflammasome-associated caspases in disease progression (Sun and Scott 2016). Among the evaluated inhibitors, Ac-YVAD-CHO was actively evaluated against endotoxaemia in male Sprague-Dawley rats, where selective inhibition of caspase-1 demonstrated promising anti-inflammatory effects during the pre-clinical stage (D’Lima et al. 2006; Boost et al. 2007).

Similarly, Z-YVAD-FMK, a peptide-based irreversible inhibitor reported by Bian et al. (2009) targeting both caspase-1 and caspase-4, exhibiting therapeutic potential in the inflammatory conditions and progressing through pre-clinical investigations. Another study conducted by Chauvier et al. (2007) used Q-VD-OPh to target broad-spectrum caspases, including caspase-1, followed by -2, -3, -6, -8 and -9 respectively. This inhibitor generally demonstrated neuroprotective potential in traumatic brain injury (TBI) and spinal cord injury (SCI)-induced rodent models, suggesting that simultaneous suppression of inflammatory and apoptotic caspases may provide therapeutic benefits in traumatic central nervous system injuries.

Among the clinically advanced compounds, VX-765 (named Belnacasan), an orally bioavailable prodrug, exhibited potent and selective inhibition of caspase-1 (MacKenzie et al. 2010). VX-765 progressed to phase Ⅱ clinical trials for the treatment-resistant epilepsy and inflammatory complications. Likewise, VX-740 (Pralnacasan) is a peptidomimetic inhibitor investigated by Poreba et al. (2013), an orally active caspase-1 inhibitor developed by Vertex Pharmaceuticals for the treatment of rheumatoid arthritis (RA) and osteoarthritis (OA). VX-765 demonstrated potent inflammatory activity, thereby suppressing IL-1β maturation; however, its phase Ⅱ clinical trials were discontinued due to its liver toxicity observed in long-term animal studies, despite the absence of severe hepatotoxicity in humans. In sepsis research, Poreba et al. (2015) reported that VX-166, a multi-caspase-1 inhibitor targeting caspase-1, -3 and -7 was evaluated in cecal ligation and puncture (CLP)-induced murine models. This inhibitor exhibited protective effects by simultaneously suppressing inflammatory and apoptotic pathways, highlighting the therapeutic relevance of combined caspase inhibition during systemic inflammatory responses.

**Table 3:** Summarized potential inhibitors reported for caspase-1 in clinical and pre-clinical trials

Diseases	Drug name	Targets	Animal models	Clinical trials	References
Endotoxaemia	Ac-YVAD-CHO	Caspase-1	Male Sprague-Dawley rate	Pre-clinical stage	D’Lima et al. (2006); Boost et al. (2007)
Inflammation	Z-YVAD-FMK	Caspase-1 and -4	.	Pre-clinical stage	Bian et al. (2009)
Traumatic central nervous systems injuries	Q-VD-Oph	Caspase-1, -2, -3, -6, -8, and -9	TBI-induced rodents and SCI-induced rodents	…	Chauvier et al. (2007)
Psoriasis	VX-765 (Belnacasan)	Caspase-1	…	Phase Ⅱ trial	MacKenzie et al. (2010)
OA and RA	VX-740 (Pralnacasan)	Caspase-1	Collagen-induced mice SCW-induced mice	Phase Ⅱ trial	Poreba et al. (2013)
Sepsis	VX-166	Caspase-1, -3, and -7	CLP-induced mice	…	Poreba et al. (﻿^2015^)

Where “…” indicates not reported and traumatic brain injury (TBI)

### Prospects and recommendations

There are still many areas that require additional investigation to improve the understanding and treatment options for caspase-1 in inflammation. It is necessary to have significant knowledge of the exact molecular mechanisms that control the activation and regulation of caspase-1 within the inflammasome. Cryo-electron microscopy and single-cell studies offer extensive structural information that may aid in the identification of novel regulatory sites and interaction pathways involved in inflammation. Furthermore, examining the relationships between different inflammatory signaling pathways and caspase-1 may reveal new regulatory networks and potential therapeutic targets to inhibit caspase-1. In order to better our understanding of the complicated in vivo interactions between caspase-1 and its opponents, we plan to develop and utilize enhanced models that involve human organoids and animals. It is important to conduct additional investigations towards the development of safer, more effective, and selective inhibitors for caspase-1, incorporating both naturally isolated from plant sources, naturally derived, and synthesized compounds. The advancement of these inhibitors aimed at targeting various phases of caspase-1 activation presents significant potential for tackling more serious diseases. Evaluating the safety and efficacy of these new ligands in animals as well as in humans will support the development of innovative treatments for inflammatory diseases.

To fully make use of these wonderful opportunities, scientists and medical professionals must ensure that they extend their interdisciplinary cooperation to include specialists from structural biology, biochemistry, molecular biology, clinical medicine, and other related areas. This combined work will help to develop new caspase-1 inhibitors and investigate their medicinal uses that require new research facilities and methods to comprehend caspase-1 and find inhibitors. One of the ways in which the translation of breakthrough scientific research into effective new medical treatments can be made faster is by making it easy for collaborations to be formed between the public sector and the business sector. It is very necessary to set up standard procedures and guidelines to ensure that caspase-1 inhibitors are used in a manner that is both effective and safe in the clinics.

## Conclusion

Caspase-1 has emerged as a pivotal regulator of inflammation and pyroptotic cell death through its critical role in the maturation of IL-1β and IL-18, positioning it as the center of numerous inflammatory signaling pathways. Abnormal regulation of caspase-1 is strongly linked to a wide range of chronic inflammatory and autoimmune diseases, as well as neurodegenerative disorders, metabolic syndromes, and cardiovascular complications, highlighting its significance as a promising therapeutic target. In this review, we comprehensively explored the mechanistic basis of caspase-1 activation and inhibition by integrating evidence from computational, experimental, and preclinical investigations. A wide spectrum of inhibitors, including natural phytochemicals, synthetic molecules, and in silico-predicted compounds, demonstrated remarkable anti-inflammatory potentials, thereby modulating the NLRP3/caspase-1/IL-1β axis via in vitro, in vivo, and preclinical models. Among them, natural flavonoids, phenolic acids, and polyphenolic extracts are the most attractive candidates, thereby posing both anti-inflammatory and antioxidant effects. Synthetic inhibitors are very specific, and issues, such as selectivity, bioavailability, and immunosuppressive effects, still confound the field. Further studies are needed to underscore the potential of integrating all modules to find an effective, safe, and more selective candidate by targeting caspase-1.

## Data Availability

No datasets were generated or analysed during the current study.

## Abbreviations

Caspases: Cysteine-aspartate proteases
IL-1β: Interleukin 1beta
IL-18: Interleukin 18
NLRP3: Nod-like receptors family pyrin domain containing 3
DED: Death effector domain
CARD: Caspase recruitment domain
PPRs: Pattern recognition receptors
NLRP1: Nod-like receptor family pyrin domain containing 1
NLRP6: Nod-like receptor family pyrin domain containing 6
NLRP7: Nod-like receptor family pyrin domain containing 7
RIG-1: Retinoic acid-inducible gene 1
PAMPs: Pathogen-associated molecular patterns
DAMPs: Damage-associated molecular patterns
CDL: Caspase docking loop
IDL: Interdomain linker
ASC: Apoptosis-associated speck-like protein containing CARD
TLRs: Toll-like receptors
LPS: Lipopolysaccharide
K^+^: Potassium
Cl^−^: Chloride
Ca^2+^: Calcium
ROS: Reactive oxygen species
NEK7: Never in mitosis gene A-related kinase 7
TXNIP: Thioredoxin-interacting protein
PYD: Pyrin domain
GSDMD: Gasdermin D
SHP2: Src homology-2 domain-containing phosphatase-2
TNF-α: Tumor necrosis factor-alpha
mtc-QSAR-MLP: Multilayer perceptron neural network
NLRC4: Nod-like receptor family CARD domain-containing protein 4
MOE: Molecular operating environment
ADMET: Absorption, distribution, excretion and toxicity
DFT: Density functional theory
GSTO-1: Glutathione-S-transferase omega-1
DAB: 1, 2-diacetylbenzene
TERM-1: Triggering receptor expressed on myeloid cells 1
DAP12: DNAX-activating protein 12kDa
CAPS1: Calcium-dependent activator protein for secretion 1
IF16: Interferon-inducible protein 16
Pparγ: Peroxisome proliferator-activated receptor gamma
Srebp-1c: Sterol regulatory element-binding protein 1c
Ppar-α: Peroxisome proliferator-activated receptor alpha
UCP2: Uncoupling protein 2
HFS: High-fat-sugar
TRPV1: Transient receptor potential cation channel subfamily V member 1
FAF1: Fass associated factor 1
AMPK: AMP-activated protein kinase
HIR: Hepatic ischemia-reperfusion
adgb: Androglobin
cbr1: Carbonyl reductase 1
decr1: 2, 4-dienoyl-CoA reductase 1
eif5: Eukaryotic translation initiation factor 5
uchl5: Ubiquitin carbonyl-terminal hydrolase l5
lmo7: LIM domain only 7
ckmt2: bdhh1, creatine kinase, mitochondrial 2
cox7a: Cytochrome c oxidase subunit 7a
STZ: Streptozotocin
TXNIP: Malfunction, thioredoxin-interacting protein
AMPK: Adenosine monophosphate-activated protein kinase
HFD: High-fat diet
BUN: Blood urea nitrogen
UACR: Urine albumin to creatinine ratio
GIH: Glomerular interstitial hyperplasia
GH: Glomerular hypertrophy
Trx: Thioredoxin
(TX)NIP: Thioredoxin-interacting protein
HOMA: Homeostasis model of assessment
IR: Insulin resistance
GDM: Gestational diabetes mellitus
NALFD: Non-alcoholic fatty liver disease
Aβ: Amyloid beta protein
SCI: Spinal cord injury
DSS: Dextran sulfate sodium
UC: Ulcerative colitis
cd11b: Cluster of differentiation molecule 11b
hmgb1: High mobility group box 1
IDD: Intervertebral disc degeneration
